# Digital tools for assessing chronic pain in children (5–11 years): Systematic review

**DOI:** 10.1002/pne2.12106

**Published:** 2023-04-19

**Authors:** Amberly Brigden, Megha Garg, Mairi Deighan, Manmita Rai, Jamie Leveret, Esther Crawley

**Affiliations:** ^1^ Digital Health, School of Computer Science, Electrical and Electronic Engineering University of Bristol Bristol UK; ^2^ Bristol Medical School: Population Health Sciences University of Bristol Bristol UK; ^3^ King's Clinical Trial Unit, Institute of Psychiatry, Psychology and Neurosciences King's College London London UK; ^4^ Centre for Academic Child Health, Bristol Medical School University of Bristol Bristol UK

**Keywords:** child, chronic pain, digital tools, pain measurement, pediatrics

## Abstract

Pediatric chronic pain places a significant burden on children, their families, and healthcare services. Effective pain measurement is needed for both clinical management and research. Digital pain measurement tools have been developed for adult and adolescent populations however less is known about measurement in younger children. In this systematic review, we aimed to identify, describe, and evaluate (in terms of acceptability) digital tools for the assessment of chronic pain in children (5–11 years). We searched five databases (Cochrane Library, EMBASE, MEDLINE, PsycINFO, and CINAHL), between January 2014 and January 2022. We included empirical studies which included digital tool/s to assess pain in children aged between 5–11 years with chronic pain conditions. We independently double‐screened the papers to determine eligibility. We followed PRISMA guidelines for reporting. A total of five papers, covering four digital tools, were included. The digital tools used ranged from a static online survey to a highly interactive, personalized tablet application. Two studies were cross‐sectional and two collected longitudinal pain data via electronic devices outside the clinical setting. Digital features of the tools included: dynamic testing (*n* = 2), notifications/prompts (*n* = 1), data transmission (*n* = 1), remote monitoring (*n* = 1), accessibility (*n* = 1), data visualization/feedback (*n* = 1), personalization/customization (*n* = 1), gamification (*n* = 1) and data labeling (*n* = 1). Qualitative usability data was only available for one of the tools, which indicated its acceptability and highlighted preferred features/functions by child users (creative and personalizable features, gamification features), and parental users (symptom tracking). This review has highlighted the limited number of digital assessment tools available for children with chronic pain aged 5–11. This review identified some examples of technology enabling the capture of longitudinal, repeated measurement of multiple dimensions of pain (intensity, location, quality). We suggest directions for future research.

## 1 INTRODUCTION

Pediatric chronic pain is highly prevalent and places a significant burden on the child, their family, and healthcare services.^1^, ^2^ Effective assessment and measurement of pediatric chronic pain are needed to inform clinical decisions^3^ and enable high‐quality research (trials and epidemiological studies). However, assessment/measurement of pain in children is complex due to the developmental constraints of this younger age group.^4^

Pain is primarily a subjective, internal phenomenon, and so the gold standard for pain assessment is a self‐report of the individuals pain experience.^5^ Adaptations for child self‐report include pictorial methods such as faces scales,^6^ and color analog scales,^7^ as well as physical methods such as the pieces‐of‐hurt scale/poker chips method.^8^ There are however problems with the accuracy of these measures given that they are subject to cognitive biases (e.g. recency effects, emotional salience, and recall bias),^9^ and are limited in the quality of data collected. They are also typically administered at static/distal timepoints,^10^ which gives limited insight into the experince of pain over time. Further, the measures are unidimensional, reducing the experience of pain to a single number.^11^ Multi‐dimensional measures of pain (capturing location, quality, and affective response) have been developed for adult population,^12^ but are inappropriate for younger populations; with lengthy questions and long lists of adjectives to describe pain, beyond the reading, and comprehension abilities of younger children. New approaches are needed to improve the accuracy and quality of pain measurement for younger populations.

Innovative digital approaches may improve the accuracy of pain measurement by enabling the capture of repeated, “in the moment” and “in the context” pain data, known as ecological momentary assessment (EMA). EMA improves accuracy by reducing cognitive biases^13^, ^14^ and imporving ecological validaity.^15^ The repeated nature of EMA enhances the quality of data by offering insights into change over time and illness fluctuations.^15^ Digital EMA has received limited attention from children under 7 years.^16^

Digital approaches may also enable better quality assessment with the use of interactive, visual features to capture multidimensional assessment of pain, including quality, intensity, and location of pain. For example, Pain‐QuILT^17^ uses icons to represent the quality of pain (e.g. a matchstick for “burning pain”), with interactive body‐mapping to illustrate the pain location (“dragging‐and‐dropping” pain icons to the specific area of the body) and *painimations* ^18^ are digital animation‐based pain assessment tools. Neither of these tools have been validated for use with younger populations.

As well as improving data capture, digital technologies can analyze and report data in real‐time to. A systematic review of pain Apps found that the majority of apps included data feedback to users, for example interactive calendars allowing users to view summaries of results and graphical methods showing details of the pain episodes.^19^ Research focused on adult populations has explored the potential of improving clinical decision‐making and pain management by making use of platforms which visualize longitudinal pain data sets.^20^

### 1.1 Aims

This systematic review focused on digital tools to aid the assessment of children (5–11 years) with chronic pain. The aims were to identify, describe, and evaluate the acceptability^21^ of the digital pain assessment tools.

## 2 METHODS

We pre‐registered our systematic review protocol on PROSPERO (CRD42020165037).^22^

### 2.1 Search and screening procedures

Our search strategy (Appendix A), developed with a data specialist, included keywords and MeSH headings for (1) children aged 5–11, (2) chronic pain, (3) pain assessment (4) digital approaches. We searched five databases, between January 2014 and January 2022: The Cochrane Library, Ovid EMBASE, Ovid MEDLINE, Ovid PsycINFO, and CINAHL. We also searched Gray literature^23^ and the reference lists of eligible articles.^24^ We exported identified articles into EndNote and performed deduplication. We used the software Rayyan and Covidence to independently double‐screen articles against the eligibility criteria outlined in Table 1, at the level of title and abstracts (stage one) and then the full‐text articles (stage two). Reasons for exclusion were recorded at stage two. Discrepancies at both stages were discussed and resolved in meetings by reviewers. If unable to locate full‐text articles at the second stage of screening, we attempted an inter‐library loan and contacted the author/s to request the full text. We manually checked the reference lists of eligible articles to identify any additional eligible articles.

**TABLE 1:** Study eligibility criteria.

Eligibility criteria	Details
Participants	
Condition	Chronic pain and chronic conditions with pain as a symptom, either self‐reported or clinically diagnosed
Age	Children aged between 5–11 years of age (this review aimed to examine digital interventions for children in the developmental stages of middle childhood). Also, studies including children belonging to a wider age range (e.g., 4–16 years), provided the data were stratified by our age of interest thus allowing data from this specific age group to be extracted
Digital pain assessment	
Pain measurement	Pain outcome measure/s or assessment tool/s completed by the child or by a proxy (for example parents, guardian, or other caregivers at home)
Digital administration	The outcome measure/assessment tool being administered digitally, including those delivered via the internet (static or interactive websites, automated emails, or web‐based apps), personal computers (PCs; e.g., PC videogames), social media, mobile phones (automated phone calls or short text messages), or smartphones (mobile websites or smartphone apps)
Study characteristics	
Study design	Empirical study designs
Study date	The search was restricted to studies published after January 2014 until January 2022, as digital tools undergo rapid development changes and older interventions become clinically irrelevant^25^
Other	Available in the English language and published in a peer‐reviewed journal

### 2.2 Data extraction

We independently double‐extracted the following data from included articles, using a self‐designed data extraction tool:

1. *Study characteristics*: aims, study design, and sample size.
2. *Participant characteristics*: age and health condition.
3. *Characteristics of the digital pain measurement tool*: the type of digital device, digital features, the pain measure, respondents (child or proxy), frequency, and time points.
4. *Quantitative evaluation of acceptability*; including response rates to the measure at the identified time points.
5. *Qualitative evaluation of acceptability*: data from qualitative interviews or focus groups with child users, their parents/carers, or clinicians. We planned to use meta‐ethnography to synthesize qualitative data on acceptability.

We resolved any conflicts by discussion among the reviewers. If no consensus was reached, the final decision was taken by a third independent reviewer.

### 2.3 Data synthesis and analysis

We synthesized studies using a narrative synthesis to describe and evaluate the digital tools. This approach allowed data synthesis from heterogeneous studies.^26^

## 3 RESULTS

**FIGURE 1:**
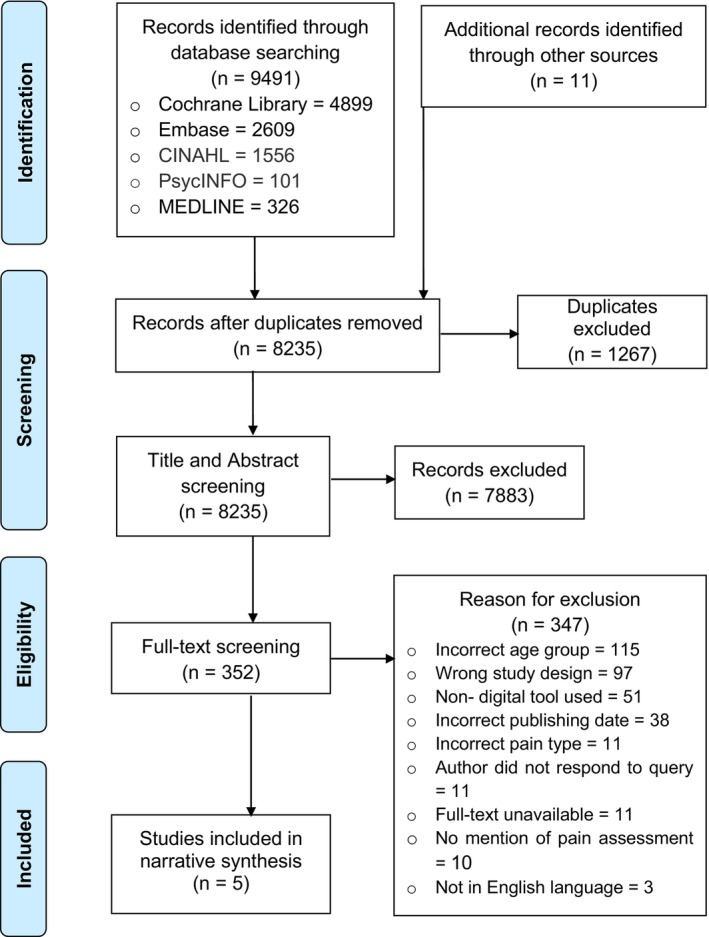
PRISMA 2009 flow diagram.

The search identified 9502 papers, 8235 after de‐duplication. In total, 7883 were excluded at the title and abstract review stage, and 347 were excluded at the full‐text review stage. Five papers, describing four different digital tools, were eligible for the inclusion in our review. See Figure 1 for the PRISMA^27^ diagram.

We found one digital tool which included participants 1 year older than the planned inclusion, i.e., 5–12 years. Due to the low number of papers identified, and 12‐year‐olds still being defined as middle childhood,^28^ we included this paper.

### 3.1 Study and participant characteristics

Two studies were longitudinal. One was a randomized control trial (RCT) assessing the efficacy and safety of Prasugrel in reducing the rate of vaso‐occlusive events in children with sickle cell anemia. The study aimed to capture electronic patient‐reported outcome diary (ePRO) completion rates and compliance in children and examine factors contributing to diary completion rates and compliance. The trial included 311 children, out of which 132 were aged between 6–11 years.^29^ The second longitudinal study was a feasibility trial, with a sample size of 19, which sought to describe the self‐reported pain experiences of children with cancer, aged 6–12 years, using the *Color Me Healthy* app.^30^ We also identified a linked paper; a mixed‐methods study exploring the feasibility and acceptability of the *Color Me Healthy* app.^31^

Two studies were cross‐sectional. One was a descriptive cross‐sectional survey aiming to investigate the relationships between pain intensity, pain anxiety and behavioral and emotional problems in children with cerebral palsy using an online survey accessed via an email link to capture pain intensity. This study analyzed data from only 61 participants, 39 of whom were aged between 5–11 years.^32^ The second was a retrospective, cross‐sectional study, with a sample size of 92, aiming to investigate the mental, physical, and psychosocial impact of weight‐bearing restrictions on children aged 5–7 years with Perthes Disease, using PROMIS to capture pain interference.^33^

### 3.2 Characteristics of the digital pain assessment tools

Table 2 summarizes the four digital tools identified in the review: the ePRO diary, the *Color Me Healthy* app, the online survey accessed via an email link and the PROMIS measure.

**TABLE 2:** Characteristics of the participant group and digital pain assessment tool, in order of date published (most recent first).

Ref	Digital device	Pain measurement	Respondents	Frequency and time points
Do et al. (2021)^33^	Mobile Electronic Tablet administering PROMIS. Administered in the clinical setting.	Pain interference	Parent‐Proxy only	Administered once
Bernier Carney et al. (2021)^30^, ^31^	Electronic Tablet with the *Color Me Healthy a*pp installed. Administered in the clinical setting.	Pain severity Pain location Qualitative description of pain	Primarily child self‐report	Completion for at least 5 days between clinic visits/inpatient admissions.
Heath et al. (2017)^29^	ePRO diary; an electronic hand‐held device with touch screen and stylus. Administered outside of the clinical setting.	Pain intensity Pain interference	Child self‐report	Twice daily for 9 months
Yamaguchi et al. (2014)^32^	Email with link to a survey accessible on computer/smartphone. Administered outside of the clinical setting.	Pain intensity Pain frequency Pain anxiety	Option of child self‐report or parent‐proxy	Administered once

#### 3.2.1 Digital devices

For three of the digital pain tools, participants were provided with the digital equipment to complete the pain assessment/s. Of these, one of the tools was an electronic tablet device which children used in a clinical setting. The tablet had the PROMIS application preloaded for children to use. The other two tools, the ePRO diary and the *Color Me Healthy* app, were used outside of the clinical setting. The *Color Me Healthy* app was preloaded onto an electronic tablet device for the children to use. The ePRO diary was an electronic ‘handheld device with a touch screen and stylus’. The fourth study sent participants an email containing a link to an online survey which could be accessed on a computer or smartphone.

Two of the digital devices, the ePRO diary and the *Color Me Healthy* app, had training/on‐boarding procedures for the digital devices and the app features. *Color Me Healthy* app had been loaded to use during the data collection period. Children were assisted to set up a user account and were oriented to the app and its features. The ePRO diary was setup by study site personnel and participants were trained on the device using the Patient Quick Start Guide which summarizes basic functions of the diary. The diary had a practise mode to allow participants to be trained on correct use. Participants were also given written diary instructions and the study site personnel had access to an optional training video to provide additional training.

#### 3.2.2 Digital features

*Dynamic testing*: Both the ePRO diary and the PROMIS survey used adaptive testing methods. For the PROMIS survey, participants given the questionnaire before 2018 completed computer adaptive test measures. The ePRO diary added an extra question to the 5‐item questionnaire based on the patient's previous response.

*Notifications/prompts*: The ePRO diary included built in notifications and alarms to prompt data entry. The alarms were disabled once the child had entered data.

*Data transmission*: The ePRO diary data was wirelessly transferred to a central database each evening. For users with unreliable wireless connection at home, data could be uploaded during a scheduled site visit.

*Remote Monitoring*: The ePRO diary allowed both the research team and the study sponsors to remotely monitor user data. Via the study website, the research team could access information for each participant, such as ePRO diary battery level, diary data, and compliance data. The study sponsor could access enrolment data and compliance summaries.

*Accessibility*: The ePRO diary could be translated into 11 languages.

*Data visualization/feedback*: The *Color Me Healthy* app had a feature to present data back to users. Allowing users to track and reviews trends in symptom severity and distress.

*Personalization/customization*: The *Color Me Healthy* app allowed users to customize the apps homepage and design their own avatars. Via the customizable avatar children identify their symptoms using drawing features, brief checklists, and short‐answer responses. The app also included a sketchpad to allow children to be creative and reflective.

*Gamification*: The *Color Me Healthy* app uses a reward‐based system to encourage data entry. Children receive one award for logging in and a second award for completing a daily health promotion activity. The activities were listed on a goals page within the app.

*Data Labeling*: The ePRO diary created separate log‐in details for the caregiver and child to identify who had entered the data.

#### 3.2.3 Pain measurement

*Pain intensity/severity*: Three studies included a measure of pain intensity or severity, where the intensity/severity score was reduced to a single number. The ePRO pain diary and the email survey both captured pain intensity using faces pain scales; The Faces‐Pain Scale revised (FPS‐R, six faces aligned on a horizontal line depicting expressions from “no pain” to “most pain possible”) and The Wong‐Baker Faces Pain Rating Scale (W‐B FPRS, a six‐point visual Likert‐type scale including six different faces that range from “no pain” to “worst pain”) respectively. The email survey offered an alternative pain intensity measure for non‐communicative children; the Non‐Communicating Children's Pain Checklist‐Revised (NCCPC‐R, which assesses pain via a parental behavioral observation of children's vocal cries, social behaviors, facial‐expressions, and physiological signs). In the *Color Me Healthy* app, children were asked to indicate the presence of pain via a checklist. If present, children were asked to rate pain severity and “bother” with rating scales ranging from “none” to “a lot”. The term bother was used as a developmentally appropriate expression to indicate the level of distress a child may experience.

*Pain interference*: Two studies captured pain interference; the degree to which pain interferes with individual's daily activities, or affective domain of pain,^34^ indicated as a single number. The ePRO pain diary captured interference in addition to intensity, capturing interference via a 5‐item questionnaire. The PROMIS measure captured interference only, via the PROMIS Parent‐Proxy Short‐Form Version 2.0 Pain Interference/Parent‐Proxy short‐Form version 2.0 Pain interference 8a. This measure involved computer adaptive testing; a dynamic form of testing that optimizes the item bank by using the respondents' previous responses to generate the next question, reducing the number of questions needed to provide an accurate T‐score. The measure includes eight questions, prefaced by “in the past 7 days, …” parents or guardians of patients answer each question based on a 5‐point Likert scale ranging from “never” to “almost always”. This paper did not provide any further detail about the nature of the pain measurement (i.e., intensity, location, quality, etc.).

*Pain frequency*: The cross‐sectional email survey included a measure of pain frequency over the last 4 weeks, measured using a single‐item question. The measures used in the longitudinal study designs, the ePRO pain diary and the *Color Me Healthy* app, did not explicitly include an item on pain frequency, but the repeated measurement over a longitudinal period meant that frequency was inherently captured.

*Pain location*: The *Color Me Healthy* app, enabled children to localize pain symptoms to specific areas of the body. Children selected pre‐defined region/s of the body on their avatar and then used a drawing feature to indicate the location of the symptom.

*Pain anxiety*: The email survey, included the 23‐item Fear of Pain Questionnaire – Parent report (FOPQ‐P).

*Qualitative description of pain*: The *Color Me Healthy* app included a feature which enabled children to provide qualitative descriptions of their pain. Although not specifically directed toward pain, the symptom assessment feature includes four brief questions to which children could provide a free‐text response. The questions included: How are you feeling today? What is the best thing about today? What is bothering you the most today? Did anything else make you feel sick today? The app also featured a diary in which children had the option to write about their day.

#### 3.2.4 Respondents

*Child self‐report only*: The *Color Me Healthy* app was designed primarily for child self‐report. Parents were instructed to allow the child to be as independent as possible in responding to items within the app. Parents of children with early/limited reading skills were permitted to support their child in reviewing items within the app (parent‐assisted completion). The ePRO pain diary subjective data questionnaire (level of pain intensity and interference with daily activities owing to sickle cell–related pain) was for child self‐report, although caregivers could enter the children's responses into the diary (parent‐assisted completion).

*Parent‐proxy only*: Both the PROMIS Pain Interference measures and the email survey were parent‐proxy reports only.

#### 3.2.5 Frequency and time points

*Longitudinal*: The ePRO pain diary collected pain data twice daily for 9 months. For the *Color Me Healthy* app, children were asked to use the app for at least 5 days between scheduled ambulatory clinic visits or inpatient admissions.

*Cross‐sectional*: Both the PROMIS pain interference measures and the email survey were cross‐sectional studies and therefore only measured pain at one time point.

### 3.3 Evaluation of feasibility and acceptability

#### 3.3.1 Qualitative data

The study exploring the feasibility and acceptability of the *Color Me Healthy* app^31^ reported qualitative data from semi‐structure interviews on the acceptability of the app (*n* = 18 children, 19 parents). Most children (*n* = 16) stated they would be willing to use the app again and all children identified areas and features of the app they enjoyed. Children enjoyed features that allowed them to be creative and personalize the app such as the sketchpad, diary entries, and avatar creation. Ten respondents enjoyed the gamified features of the app and the use of rewards as incentives. Parents valued the ability for children to self‐report their symptoms, reporting that this enabled greater awareness of the child's symptoms, facilitated conversations between the parent and child about their symptoms and had potential for more accurate and precise symptom tracking over time. Both children and parents supported the use of the app to track symptoms and highlighted the potential clinical benefits Including assisting communication at hospital appointments, and symptoms being “easier to explain” when using the app. Suggestions for app improvements referred to usability (reduced loading times and improve navigation) and enhancing user engagement (more activities and widening the gamified features). Parents expressed an interest in being able to monitor their child's use of the app possibly via a parent access account or paired application.

#### 3.3.2 Quantitative data

*Longitudinal studies*: The highest completion rates for the longitudinal ePRO pain diary, was for children aged 6 to <12 years. In this group, completion rates for the 9‐month diary collection period were 96.2% ± 6.4% (calculated as the number of daily diary entries divided by the total number of expected daily diary entries). In the *Color Me Healthy* app study^31^ Children were advised to use the app for at least 5 days between clinical visits. All children used the app at least 1 day with total days of use ranging from 1 to 12 days (median 4 days). Nine children used the app 5 days or more, and another four children used the app for 4 days. Children most frequently used the symptom‐reporting page and the drawing page.

*The cross‐sectional studies* only reported the number who had completed a pain score at one time point. For the PROMIS study, pain interference score was available for all 92 participants, information about missing items was not provided. For the online survey, 352 parents/guardians of children were emailed out, with a total of 77 participants completing the survey. 32 surveys were excluded from analysis due to being incomplete.

## 4 DISCUSSION

### 4.1 Summary

This is the first systematic review to identify, describe, and evaluate the acceptability of digital tools for the assessment of chronic pain in children (5–11 years). This review highlights the limited number of tools available for this younger population; we only identified four. There was wide variation in the technology used and the extent to which the digital tools harnessed technological capabilities. On the more sophisticated end of the technology spectrum was the ePRO diary (repeated pain measurement enhanced through prompts/notifications and an ability to transmit data remotely to a central database) and the *Color Me Healthy* app (repeated pain measurement including multidimensional assessment, gamification, and personalization), with response rates and qualitative data (*Color Me Healthy* app only) indicating the acceptability of these tools. On the lower end of technology sophistication was a static online survey, which had a response rate of 22%. There was diversity in the dimensions of pain that were measured (pain intensity/severity, pain interference, pain location, qualitative description of pain, pain anxiety, pain frequency) with the most common being intensity/severity and interference.

### 4.2 Implications in the context of existing literature

This review highlights the research gap for digital pain assessment tools for the 5–11 year age group, in comparison to the large number of tools and range of innovations available for older populations (adults and adolescents).^17^, ^18^, ^35^ This is typical of digital health technologies being under researched in pediatric populations.^36^, ^37^ From the limited number of tools identified, there were examples of innovation that may improve pain data capture and data output/usage in the future. These are discussed below along with recommendations for future research.

#### 4.2.1 Improving the accuracy and quality of pain data via digital EMA

Both the *Color Me Healthy* app and the ePRO diary used an ecological momentary assessment (EMA) approach; taking repeated measurement of pain in the child's context. EMA reduces cognitive biases^13^, ^14^ improves ecological validity^15^ and offers insights into change over time and illness fluctuations.^15^ Indeed, qualitative data from the *Color Me Healthy* app indicated that users felt the tool had the potential for more accurate and precise symptom tracking over time. The tools in this review used digital features to enhance EMA data capture such as integrating notifications and alarms to improve response rate.^38^ As a limitation, these digital EMA tools used devices which were not readily available outside the research context (ePRO diary) and non‐portable (tablets). For adult and adolescent populations, digital EMA has harnessed the ubiquitous availability and portability of smartphones, to improve easy‐to‐use, repeated, and ecological measurement. This also offers the ability to capture momentary assessment of variables such as environmental conditions, GPS, physical activity, and biophysical signals (such as heartrate) over time to enhance the understanding of pain experience in everyday life.^39^ There is no such research in younger populations and could be an avenue for future research.

#### 4.2.2 Improving the quality of pain data via digital multi‐dimensional assessment

Two of the studies captured pain intensity and/or interference only. Unidimensional pain assessments reduce pain levels to a single number, failing to provide a complete picture of the child's pain. Ideally, pain assessment involves the measurement of multiple domains such as intensity, location, quality, duration, frequency, and affective response^35^, ^40^ The online survey included an emotional dimension (pain anxiety), and the *Color Me Healthy* app included measures of intensity, location, and free‐text qualitative descriptions. Text‐based and free‐recall responses may not be suited to the developmental capacities of younger children, and free‐text responses are hard to analyze for research. Future directions of digital tool development could focus on exploring appropriate methods to capture pain quality, such as using visual icons^17^ or animations.^18^

#### 4.2.3 Improving pain data output through real‐time digital transmission and feedback

The digital features of the identified tools enabled data to be used in real‐time, to produce reports for the patient users and to transmit data to clinical/research teams. The *Color Me Healthy* app enabled children to track and review trends in symptom severity and distress. Parents felt this enabled greater awareness of the child's symptoms and facilitated conversations between the parent and child about their symptoms. In terms of utility for clinical teams, both children, and parents highlighted the hypothetical/potential clinical benefits of The *Color Me Healthy* app in assisting communication at hospital appointments, and symptoms being “easier to explain”. None of the tools in this review explored the potential of transmitting real‐time data to clinical teams. This type of remote clinical monitoring can improve the continuity of care for children and their families^41^, ^42^ and is worthy of exploration, with attention paid to the ethical implications of handling sensitive pediatric pain data, considering issues of data security and protocols for responding to clinical data which need immediate/quick responses. In terms of research utility, the ePRO diary transmitted data daily to a centralized database, enabling the researchers to remotely monitor patient data.

#### 4.2.4 Optimizing the user‐experince

Ultimately good data capture relies on children meaningully engaging with the self‐report measures; technology acceptability. This relates to the ease of use and the perceived usefulness^35^ and is key to enhancing adoption and sustained usage of the technology. This review highlighted features which may make tools more accetable and potentially engaging for users. The qualitative findings indicated that users of the *Color Me Healthy* app enjoyed the gamified and reward features and the ability to be creative and personalize the app. Parents expressed an interest in being able to monitor their child's use of the app. Equally, providing output from the data for children, parents/carers, and clinical teams, which can be used in a clinically meaningfuly way, needs to be designed with input from these stakeholders. Both the ePRO and the *Color Me Healthy* app including training and on‐boarding procedures, which may have enhanced the user‐experience. Further research could explore optimal ways to visualize complex longitudinal pain data.

### 4.3 Strengths and limitations

We used robust systematic review methodology, preregistering our protocol on PROSPERO, conducting independent double‐screening, and double‐extraction, and following narrative synthesis guidelines.^26^ We did not limit our review to one type of chronic pain; spanning a broad range of pain‐related health conditions enabled us to identify any tool that could benefit pain assessment, no matter the condition. We restricted the search to studies published from 2014 to ensure relevance, given the rapid pace of change in the digital health field.^25^

Studies spanning a wide age range (for example 5–18 years) were excluded if the data were not stratified by our age of interest. We would have identified a larger number of studies if we had a wider age range. However, this approach enabled us to focus on measures that were specifically aimed at our developmental age‐group of interest (middle childhood). When extracting digital features, we only extracted data on features that were explicitly reported, no inferences were made. Some digital tools may therefore have additional digital features that have not been reported in this review.

## 5 CONCLUSIONS

This review has highlighted the lack of research into digital pain assessment tools for children with chronic pain aged 5–11; we identified only four tools. Future areas of research could include exploration of smartphone and smartwatch technology to capture pain data in context along with other useful variables; investigating developmentally sensitive methods of capturing pain quality data; and exploring how to feedback pain data to key stakeholders in a clinically meaningful way. User‐involvement process, such as co‐design and Human‐Computer Interaction approaches, will aid the development and evaluation of tools which are acceptable, engaging, and clinically useful.

## AUTHOR CONTRIBUTIONS

AB conceptualized the study, supervised the study, contributed to data collection, and management contributed to the draft of the manuscript. MG led to data collection, management, and analysis, and wrote the first draft of the paper. JL, MR, and MD contributed to data collection, management, and analysis, and revised the manuscript critically, making important intellectual contributions. EC supervised the study and revised the manuscript critically, making important intellectual contributions. All authors gave approval of the final manuscript.

## CONFLICT OF INTEREST STATEMENT

The authors do not have any conflicts of interest to declare.

## Supporting information


Data S1.
