# Supplementary material for: Digital tools for assessing chronic pain in children (5–11 years): Systematic review
Source: Paediatr Neonatal Pain. 2023 Apr 19;6(3):89–97. doi: 10.1002/pne2.12106 (PMC11514301; doi:10.1002/pne2.12106)
Supplement: Supplementary file 1 — Data S1. [file PNE2-6-89-s001.docx]

**Supplementary Material:**

**EMBASE SEARCH STRATEGY**

| 1. child/ |
| --- |
| 2. preschool child/ |
| 3. child*.ti,ab. |
| 4. pedia*.ti,ab. |
| 5. primary school child*.ti,ab. |
| 6. elementary school child*.ti,ab. |
| 7. boy*.ti,ab. |
| 8. girl*.ti,ab. |
| 9. pre-adolescen*.ti,ab. |
| 10. preadolescen*.ti,ab. |
| 11. early life.ti,ab. |
| 12. kindergarten.ti,ab. |
| 13. junior school.ti,ab. |
| 14. (young adj child*).ti,ab. |
| 15. exp pain/ |
| 16. exp chronic pain/ |
| 17. exp pain measurement/ |
| 18. exp questionnaires/ |
| 19. predictive value of tests/ |
| 20. (patient adj profil*).ti,ab. |
| 21. neuropathic pain questionnaire*.ti,ab. |
| 22. paindetect.ti,ab. |
| 23. id pain.ti,ab. |
| 24. numeric rating scale*.ti,ab. |
| 25. visual analogue scale*.ti,ab. |
| 26. chronic pain grade.ti,ab. |
| 27. brief pain inventory.ti,ab. |
| 28. pain index.ti,ab. |
| 29. cellular phone.mp. or cell phone/ |
| 30. smartphone/ |
| 31. text messaging/ |
| 32. internet/ |
| 33. social media/ |
| 34. mobile applications/ |
| 35. computers/ |
| 36. software/ |
| 37. electronic mail/ |
| 38. video games/ |
| 39. digital.ti,ab. |
| 40. email.ti,ab. |
| 41. television.ti,ab. |
| 42. tv.ti,ab. |
| 43. electronic mail.ti,ab. |
| 44. new media.ti,ab. |
| 45. social media.ti,ab. |
| 46. electronic media.ti,ab. |
| 47. mobile app*.ti,ab. |
| 48. phone*.ti,ab. |
| 49. smartphone*.ti,ab. |
| 50. cellphone*.ti,ab. |
| 51. mobile*.ti,ab. |
| 52. web*.ti,ab. |
| 53. internet.ti,ab. |
| 54. app*.ti,ab. |
| 55. laptop*.ti,ab. |
| 56. video gam*.ti,ab. |
| 57. 1 or 2 or 3 or 4 or 5 or 6 or 7 or 8 or 9 or 10 or 11 or 12 or 13 or 14 |
| 58. 15 or 16 |
| 59. 17 or 18 or 19 or 20 or 21 or 22 or 23 or 24 or 25 or 26 or 27 or 28 |
| 60. 29 or 30 or 31 or 32 or 33 or 34 or 35 or 36 or 37 or 38 or 39 or 40 or 41 or 42 or 43 or 44 or 45 or 46 or 47 or 48 or 49 or 50 or 51 or 52 or 53 or 54 or 55 or 56 |
| 61. 57 and 58 and 59 and 60 |
| 62. limit 61 to yr="2014 -Current" |
